# Molecular insights into the aggregation and solubilizing behavior of biocompatible amphiphiles Gelucire® 48/16 and Tetronics® 1304 in aqueous media

**DOI:** 10.1039/d3ra04844f

**Published:** 2023-09-29

**Authors:** Deep Bhalani, Hiral Kakkad, Jignasa Modh, Debes Ray, Vinod K. Aswal, Sadafara A. Pillai

**Affiliations:** a School of Sciences, P. P. Savani University NH-8, GETCO, Near Biltech, Kosamba Surat 394125 Gujarat India sa.pillai@ppsu.ac.in; b Solid State Physics Division, Bhabha Atomic Research Centre (BARC) Mumbai 400085 Maharashtra India; c Biomacromolecular Systems and Processes, Institute of Biological Information Processing, Forschungszentrum Jülich Jülich 52428 Germany

## Abstract

A comparative analysis of the micellar and solubilizing properties of two polyethylene glycol (PEG)-based amphiphilic biocompatible excipients: Gelucire® 48/16 (Ge 48/16) and Tetronics® 1304 (T1304), in the presence and absence of salt, was conducted. As there is a dearth of research in this area, the study aims to shed light on the behavior of these two nonionic surfactants and their potential as nanocarriers for solubilizing pharmaceuticals. Various techniques such as cloud point (CP), dynamic light scattering (DLS), small-angle neutron scattering (SANS), Fourier transform infrared spectroscopy (FT-IR), UV spectrophotometry, and high-performance liquid chromatography (HPLC) were employed. The solubility of quercetin (QCT), a flavonoid with anti-inflammatory, antioxidant, and anti-cancer properties, was evaluated and the interaction between QCT and the micellar system was examined. The analysis revealed the occurrence of strong interactions between QCT and surfactant molecules, resulting in enhanced solubility. It was observed that the micellar size and solubilizing ability were significantly improved in the presence of salt, while the CP decreased. Ge 48/16 exhibited superior performance, with a remarkable increase in the solubility of QCT in the presence of salt, suggesting its potential as an effective nanocarrier for a range of pharmaceutics, and yielding better therapeutic outcomes.

## Abstract 2

Comparative analysis of PEG-based excipients Gelucire® 48/16 and Tetronics® 1304, for micellar and solubilizing properties.

## Graphical abstract

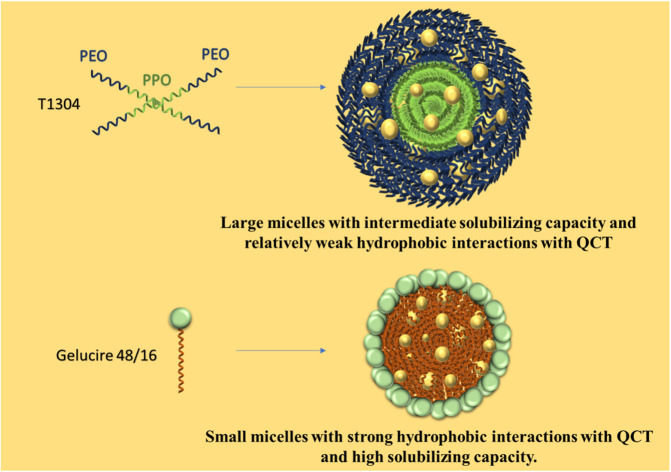

## 1. Introduction

Water insolubility of small drug molecules poses a serious challenge in their clinical applications. Currently, more than 40% of the marketed oral drugs are known to have a hydrophobic nature.^1,2^ The restricted rate of drug dissolution and poor bioavailability of drugs are some of the major drawbacks of hydrophobic drugs.^3,4^ One solution to overcome these challenges and gain the drug's therapeutic effect in the blood is by enhancing the dose of drugs. However, such high doses of the drug can give rise to a variety of other problems such as high cost of manufacturing, high toxicity, and drug formulation difficulties to name a few.^5^ Hence, over a period of time, researchers have come up with numerous techniques and drug delivery systems to overcome these challenges faced by the pharmaceutical industries.^6–8^ One of the approaches involves the use of self-assembled systems like nonionic surfactants to improve the solubilization of hydrophobic drugs by encapsulating them in the micellar core, enhancing permeability, and achieving controlled release of different drugs. The term nonionic surfactant is used to refer to both surface active agents like low molecular weight surfactants (with a neutral charge on its polar head group) such as *n*-dodecyl tetra(ethylene oxide) as well as synthetic block copolymers with high molecular weights. The blocks in synthetic block copolymers can be tailored in linear diblock (A–B), triblock (A–B–A), pentablock (A–A–B–A–A) fashion or could be arranged in branched fashion, and the micelles so formed by block copolymers are termed as polymeric micelles. However, it is important to note that, from the physicochemical viewpoint, there is a significant difference between polymeric micelles and surfactant micelles. The polymeric units get associated to form micelles at a concentration called the critical aggregation concentration (CAC), which is low in magnitude by several orders as compared to the typical surfactant critical micellar concentration (CMC) values. Further, polymeric micelles offer good kinetic stability as break down of micelles in case of low molecular weight surfactant micelles occurs in the time range of micro seconds while the polymeric micelles are known to preserve their structure for extended period of time. It is thus the better kinetic stability along with low toxicity offered by polymeric micelles, the reason for them to be preferred as compared to low molecular weight surfactant composed micelles as drug nanocarriers.^9–13^

A number of nonionic surfactants such as polysorbates, Span 20, Span 80, Cremophors®, Pluronics®, *etc.* are already in use in some commercially available solubilised oral formulations and have proven to be useful.^14^ Interestingly, some of the nonionic surfactants, besides being active solubilizers, are capable to modulate the biological response of tumor cells and modify the activity of efflux pumps associated with multidrug resistance. These micelles with adequate size, usually being more than 5.5 nm, a threshold for the renal clearance of nanoparticles, increases their circulation time in the bloodstream and tends to accumulate passively at the sites with leaky vasculature due to enhanced permeability and retention effect.^15–19^ Further, it was realized that the extracellular space in tumours is known to have lower pH as compared to the normal cells and this variation in pH is most pronounced in endolysosomal vesicles. Hence, several efforts were placed in the direction to develop pH-responsive nano vehicles to specifically release the therapeutic load at the tumour site.^20–24^ Tetronics®, a branched analogue of Pluronics®, with an additional ability to respond to pH became the most explored amphiphilic polymer and serves as a stimuli responsive nanovehicle with pH sensitive drug release.^15,25–30^ Cuestas *et al.*^31^ investigated the inhibitory effect of different concentrations of Tetronics® T904, T304, T1301 and T1107 with a wide range of molecular weights on the functional activity of three different ABC proteins, namely P-glycoprotein, breast cancer resistance protein, and multidrug resistance-associated protein, in two human hepatocarcinoma cell lines, HepG2 and Huh7 and revealed that the effect remarkably depended on the concentration and hydrophobicity of the copolymers. Cagel *et al.*^32^ in an attempt to develop potential nanocarrier for the antineoplastic drug in ovarian and metastatic breast cancer, doxorubicin found that T1107 and d-α-tocopheryl polyethylene glycol 1000 succinate (TPGS) showed better performance with high drug loading capacity and better physicochemical properties and noted that the *in vitro* drug release was more in the acidic microenvironment of tumour than in the physiological counterpart. Úriz *et al.*^33^ observed improved solubility and cytotoxicity of selenodiazoles in T904. Similarly, Puig-Rigall *et al.*^34^ developed formulations of miltefosine (MF), an alkylphospholipid for breast cancer treatment, using TPGS, T1107 and T904 and noted improved activity of MF with T904 against intracellular amastigotes. Lecot *et al.*^35^ in a recent study used T1307 modified with a 4,4-difluoro-4-bora-3a,4a-diaza-*s*-indacene fluorophore for assessing it's *in vivo* biodistribution on 4T1 tumor-bearing mice and attained results revealing promising role of T1307 micelles as radio tracer agents for breast cancer imaging. Castelli *et al.*^36^ observed the co-association of aptamer Sgc8-c-Alexa647, a biomarker in cancer with different nanostructures like F127, T1307 and pegylated liposomes. The results revealed that improved permanence was noted in circulation with T908 in tumour bearing mice as compared to free-probe. Recently, Alasmary *et al.*^37^ modified T1107 to produce Tetronic® Schiff base to improve its biological activity and disclosed that all the modified polymers showed improved anticancer activity towards MDA-MB-231 cell line.

Gelucire® 48/16 (Ge 48/16, polyethylene glycol (PEG 32) monostearate) belongs to an important class of PEG based nonionic surfactants and is known to have wide range of applications in oral and topical formulations for enhancing the solubility and bioavailability of poorly water soluble drugs^38^ as solid dispersions,^39–47^ nanoparticles,^48^ SEDDS^49^ and mixed micelles.^50^ Krawczyk-Santos *et al.*^51^ developed poly(pseudo)rotaxane(PPRs) using Kolliphor® EL, Ge 48/16 and their mixed micelles in combination with α-cyclodextrin to deliver terbinafine to nail and noted enhanced drug solubility and the results revealed the potential of using the water based formulations as topical treatment of onychomycosis. In a recent report, Krawczyk-Santos and co-workers checked the possibility for the application of PPRs for vaginal antifungal delivery and noted positive results.^52^ Arafa *et al.*^53^ explored mixed micellar systems of F127 and Gelucire® 44/14 for solubilising the drug praziquantel and observed high solubility, drug entrapment efficiency and prolonged release pattern for the optimized mixed micellar system. Shinde *et al.*^54^ explored the capacity of mixed micelles of Ge 48/16 and TPGS in solubilising hydrophobic anticancer drug, curcumin and observed improved solubility with mixed micelles than its individual components. Kushwaha *et al.*^55^ developed galactosylated Pluronics® F68 and Ge 44/14 mixed micelles to solubilize poorly water-soluble anticancer drug, harmine and targeting liver for its anticancer activity against liver cancer and revealed mixed micellar system to be an efficient nanocarrier system with improved anticancer activity and enhanced bioavailability of drug.

From the above literature survey, it can be realized that there are many reports defining the role of Tetronics® in diagnosis of cancer or as anticancer drug nanocarrier, discussing their role as single and mixed micelles. Likewise, there are several reports based on Gelucire® as lipid based nanocarrier in different physical forms and with a limited number of reports on water-based formulations. However, a study comparing the micellar and solubilising behaviour of these two classes of nonionic surfactants still lacks. Taking this into consideration, the present study aims to compare the micellar and solubilising behaviour of two nonionic surfactants, T1304 with Ge 48/16 using several techniques. The results from the present study will be highly beneficial in designing and optimization of drug formulations and guide in understanding their potential as nanocarriers for different therapeutic and pharmaceutical applications.

## 2. Experimental

### 2.1 Materials

**Fig. 1:**
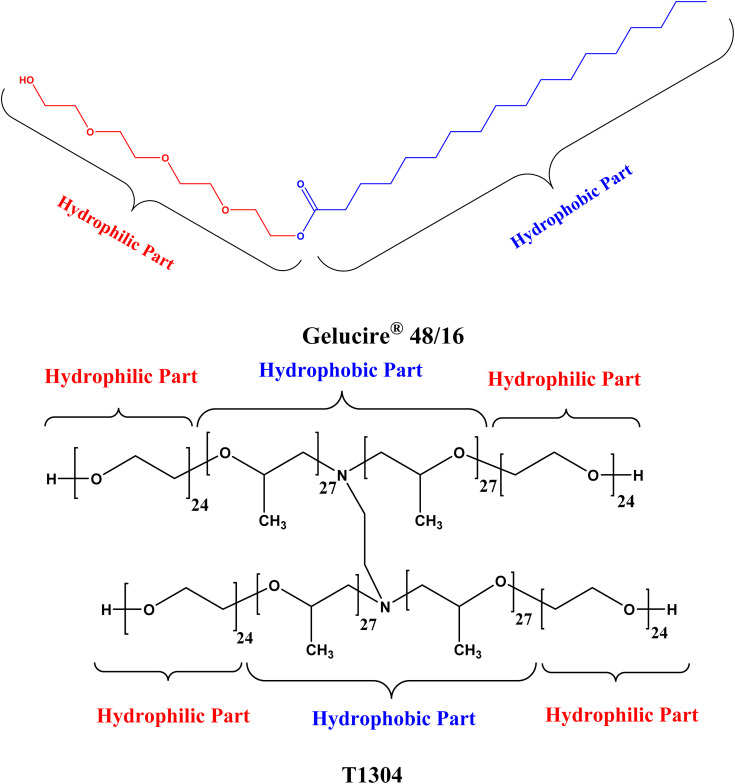
Chemical structure of Gelucire® 48/16 and T1304.

The nonionic surfactants Gelucire® 48/16 (Ge 48/16) and Tetronics® 1304 (T1304) with the chemical structure illustrated in Fig. 1 were provided as free samples by Gattefossé, India, and BASF Corp., India, respectively. The samples were used as received without any further purification. The salt, sodium chloride (NaCl) with purity ∼99% and analytical grade was purchased from Sigma Aldrich (India). The samples for measurements were prepared using deionized water from Milipore Mili-Q system except for SANS where 99.9 atom% pure D_2_O, purchased from sigma Aldrich (India), was used for the sample preparation.

### 2.2 Methods

#### 2.2.1 Preparation of stock solutions

The stock solutions for pure Ge 48/16 and T1304 were prepared by dissolving 1 g of each material in 100 mL of water, yielding 1% w/v Ge 48/16 and 1% w/v T1304 stock solutions. The weighed amount of NaCl was added to the 2 mL stock solutions of T1304 and Ge 48/16, respectively, to examine the effects of salt.

#### 2.2.2 Cloud point (CP)

The entire apparatus was heated at a rate of 1 °C min^−1^ in a temperature-controlled water bath to determine the cloud point (CP) readings for aqueous solutions. The stock solution, 2 mL, was utilised in 20 mL of glass test tubes, both with and without salt, and the thermometer was continually swirled to maintain a constant solution temperature. The first appearance of turbidity to naked eye was considered as CP. To ensure reproducibility, all of the measured CP values were taken three times. The measurements were determined to be precise with a maximum deviation range of ±1 °C.

#### 2.2.3 Dynamic light scattering (DLS)

When using DLS, one detects changes in the amount of light that is scattered by suspended particles moving in a Brownian manner.^56,57^ Plots of the autocorrelation function *vs.* time are used to display these fluctuations. Then, to determine the effective translational diffusion coefficient (*D*), these graphs are often analysed using cumulant methods. The effective hydrodynamic size (*D*_h_) of the particles is then determined using the Stokes–Einstein relation:
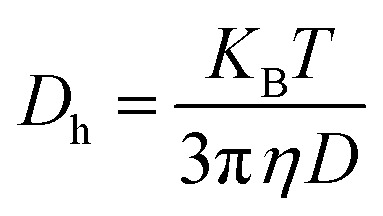
where *K*_B_ stands for Boltzmann constant, *T* is the absolute temperature, and *η* is the viscosity of the solvent.

#### 2.2.4 Fourier-transfer infrared (FT-IR) spectroscopy

The FT-IR spectra were obtained using Shimadzu FT-IR-8400S equipment. Adding two or three drops of the tested solution with KBr resulted in pellets in this case. A bulb oven was used to dry the prepared pellets overnight, and spectra were then recorded.^58^

#### 2.2.5 UV-visible spectroscopy measurements

The UV-vis spectra of both the free QCT and the QCT loaded in micellar aggregates were measured at 298 K using a spectrophotometer SHIMADZU UV-1650 PC where the samples were held in 1.0 cm quartz cuvettes. The samples were filtered and diluted with ethanol to a final ratio of ethanol to water 2 : 1 before each measurement.^59^

#### 2.2.6 Small angle neutron scattering (SANS)

The SANS experiments were carried out in the Dhruva reactor, BARC, Trombay, using a SANS diffractometer. The sample solutions were prepared in D_2_O (99.9% *D*) and the measurements were performed at 30 °C. A quartz cell with a 5 mm thickness was used to contain the solutions, and tight-fitting Teflon stoppers were used. The data were logged between 0.017 and 0.35 in terms of *Q*. For the background and solvent contributions, the observed SANS distributions were all adjusted. Using standardised techniques, the data were normalised to the cross-sectional unit.^60^

A surfactant micelle consists of a hydrophobic core encased in a hydrated shell and the hydrophobic core and the solvent have excellent contrast with one another. The scattering contrast between the hydrated corona and the solvent is anticipated to be poor due to the considerable amount of D_2_O (water of hydration) present in the outer hydrophilic corona. As a result, we assume that the form factor *F*(*Q*) simply depends on the radius of the hydrophobic core. For the case of the hard sphere potential in the Ornstein–Zernike equation, the Percus–Yevick approximation is used to derive the structural factor *S*(*Q*) of the spherical micelles in equation.^61^
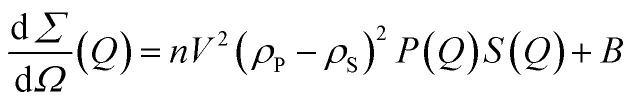


The investigation has yielded the fitting parameters, which are the micelle's mean core radius (*R*_c_), hard sphere radius (*R*_hs_), and volume fraction (*Φ*). The formula *N* = 4π*a*^3^/3*v*, where *v* is the volume of the surfactant monomer, is used to compute the aggregation number.

#### 2.2.7 High performance liquid chromatography (HPLC)

##### (A) Drug solubilization technique

Reverse phase HPLC was used to evaluate the solubilization of the drug QN (LC-2010, AHT; Shimadzu, make: Japan). ODS C18 column (250 × 4.6 mm, 5 μm particle size, 100 Å pore size; Make: Thermo scientific) was employed for the separation in the HPLC chromatogram.

The chromatographic apparatus was operated using the LC Solution software, which was also utilised for data analysis and recording. For the drug QCT, the mobile phase was made up of water, acetonitrile, and methanol (20 : 20 : 60% v/v). Also, the samples were detected utilising a UV detector for QCT at a wavelength of 262 nm. The sample injection volumes were maintained at 20 μL using a mobile phase flow rate of 1.0 mL min^−1^ and a total run period of 10 minutes. The quantification was done using a linear calibration curve with an acceptable Beer–Lambert plot based on the sample's peak area ratio of the active pharmaceutical ingredient (API) to internal standards (*R*^2^ = 0.999).

##### (B) Drug loading capacity and entrapment efficiency by HPLC

For the determination of drug loading capacity and entrapment efficiency of surfactants the drug loaded micellar solutions were prepared with varying concentration of salt (NaCl) (0, 0.15, 0.5, 1.0, 1.5 and 2.0 M NaCl) and a standard solution with QCT to compare the data. The standard solution was prepared by dissolving 5 mg of accurately weighed QCT in a pure solvent displaying complete solubility. The micellar samples were prepared using 1% w/v solution of amphiphile with calculated amount of salt. The weighed amount of QCT (5 mg) was added to all the micellar systems. After sonication for 90 min at room temperature in an ultrasonic bath, the samples were allowed to equilibrate overnight. The samples were centrifuged at 10 000 rpm for 10 min and were filtered through 0.22 μm nylon filter to remove larger undissolved part of drug or any contamination. All the samples were analysed by HPLC method. All the measurements were done at 30 °C temperature.^19,58^

The drug loading amount (DLA) and entrapment efficiency (EE%) were calculated from HPLC analysis area percentage results.
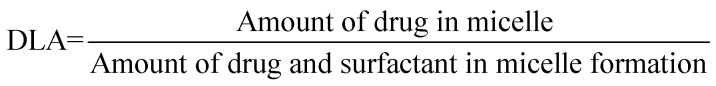

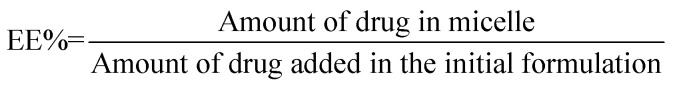


## 3. Result and discussion

### 3.1 Characterization of micelles

To comprehend and contrast the behaviour of two nonionic surfactants, Ge 48/16 and T1304, in an aqueous environment, a multitechnique approach was employed. Over the past few decades, Ge 48/16, also known as polyethylene glycol (PEG 32) monostearate, has drawn a great deal of attention for its exceptional solubilizing capabilities for pharmaceuticals with little absorption otherwise, with a special focus on solid dispersions.^41,62^ While, the extensive reports on the solubilizing behaviour of block copolymers in aqueous environment reveals the potential of T1304, a polyethylene glycol-polypropylene glycol (PEG-PPG) based star block copolymer for its excellent solubilizing capacity for poorly water-soluble drugs. Although Ge 48/16 has been the subject of numerous independent reports, little attention has been paid to how they behave in aqueous solutions. Additionally, a comparative study on two different class of nonionic surfactants, a high molecular weight PEG-PPG star block copolymer such as T1304 and a low molecular weight PEG based surfactant like Ge 48/16 is missing in reports. In order to achieve this, the current work compares the phase behaviour, micellar size and morphology, interaction with QCT molecules, and solubilizing potential for QCT of both the nonionic surfactants.

**Fig. 2:**
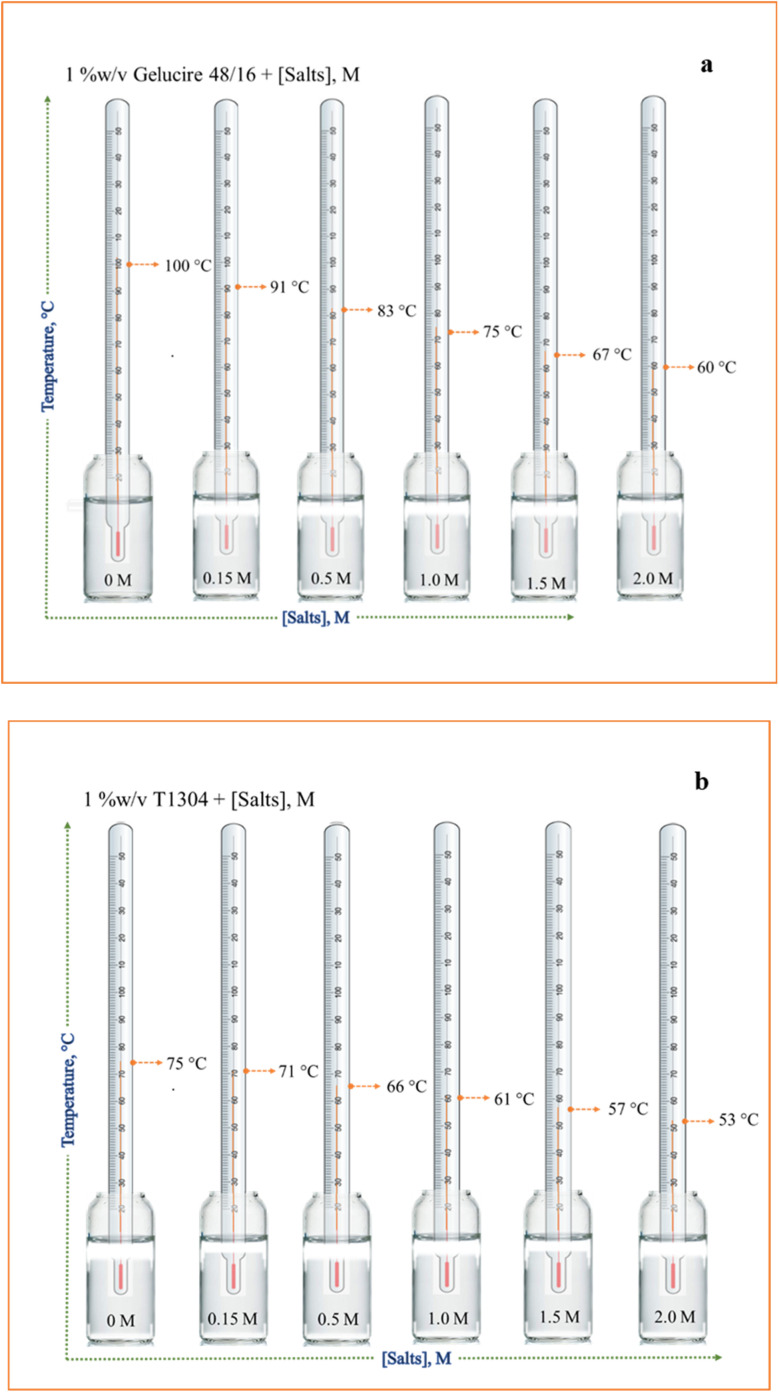
Pictorial representation of clouding phenomenon observed in 1% w/v aqueous solution of (a) Ge 48/16 and (b) T1304 as a function of salt concentration.

In order to understand the phase behaviour of both the nonionic surfactants, their cloud points were measured. The term “cloud point” (CP) refers to the temperature at which a surfactant solution that was initially clear becomes hazy due to decrease in solubility of dissolved amphiphiles at raised temperatures.^57,63^ Because a surfactant solution performs dramatically differently around the cloud point temperature, its cloud point should be taken into account when screening surfactants for certain applications. Some applications such as surfactant-mediated soil remediation required high cloud points^64,65^ while some demand lower CP values, such as cloud point extraction, which is more commonly used in two-phase regions.^65,66^ One approach to attain the desired CP value is by using the additives.^59,67^ There are several reports in literature discussing the effects of various additives, including inorganic electrolytes,^59,68^ ionic surfactants,^69^ alcohols^63,70,71^ on the clouding behaviour of different nonionic surfactants. Many researchers have reported a notable impact of salt, an important pharmaceutical excipient, on the aggregation behaviour of different nonionic surfactants.^72–75^ Though, there are reports on the clouding behaviour of PEG-PPG block copolymers in the presence of salts,^76^ it is interesting to find that there are no reports in literature discussing the clouding behaviour of Ge 48/16 and we for the first time have checked the influence of different concentrations of salt (concentrations mentioned in the Fig. 2(a) and (b)) on the clouding behaviour of Ge 48/16.

As shown in Fig. 2, the presence of salt plays an essential role in decreasing the CP for both the nonionic surfactants. In the absence of salt, the aqueous solutions of 1% w/v T1304 displayed CP at 75 °C (Fig. 2(b)), due to its moderately hydrophobic character, while the aqueous solutions of 1% w/v Ge 48/16 did not show CP up to 100 °C (Fig. 2(a)) and the solutions remained clear due to its hydrophilic nature, supporting the reported values.^30,38^ However, the addition of salt in the both the micellar solutions, significantly reduced their CP values. It has been established that, PEG-based nonionic surfactants are made up of hydrophilic and hydrophobic parts. The phenomenon of CP can be understood with the aid of hydrogen bonding. Above a certain concentration, known as the critical micelle concentration (CMC), these amphiphiles form core–shell micelles in which the hydrophobic part convolutes to form the micellar core, while the PEG chains form the heavily hydrated corona, which is primarily responsible for hydrogen bonding with water at temperatures below CP. As the temperature approaches CP, the magnitude of hydration of the hydrophilic part becomes insufficient to dissolve the residual hydrophobic moiety, eventually resulting in phase separation. In case of T1304, which is a PEG-PPG star block copolymer, the PEG and PPG blocks, exhibit varying levels of water solubility. Both the PEG and PPG block are completely soluble in water at lower temperatures. The development of micelles, where the PEG block serves as the hydrophilic shell and the PPG block as the hydrophobic core, is triggered by the decreasing solubility of PPG blocks as the temperature rises. With further increase in temperature, the solubility of PEG blocks gradually decreases until they are fully insoluble, which results in clouding and causes phase separation.^77^ For Ge 48/16, which is made up of PEG-32 (MW 1500) esters of palmitic (C_16_) and stearic (C_18_) acids, the hydrophilic component of this PEG-ester surfactant is polyethylene glycol, while the lipophilic component is fatty acid.^38^ As the temperature rises, like in the case of T1304, the hydrogen bonds established between PEG chains and water are broken. This reduces the solubility of amphiphilic units in water and leads to clouding. Nonetheless, salt has a significant impact on the composition and characteristics of water, and because sodium chloride (NaCl) helps to form the structure of water, this action lowers the CP. Likewise, the surfactant solutions experienced significant changes in CP when saturated with QCT (not shown). Specifically, the CP of pure 1% w/v T1304 aqueous solution decreased by 5 °C when saturated with QCT, which further decreased by 2 °C under saline conditions. While Ge 48/16 has a naturally high CP above 100 °C, even when saturated with QCT, its CP remained high. Similar findings were reported in relation to the influence of a poorly water-soluble drug, indomethacin, on two nonionic surfactants.^78^ The report suggests shape transitions, elevated CP, depression in dissolution temperature, and swelling in micelles saturated with indomethacin. This leads us to a conclusion that both self-assembling systems share the same mechanism for the occurrence of the cloud point phenomenon and that the solubility of the PEG chains is necessary for the solubilization of the individual amphiphile units.

It is clear from the phase behaviour that both nonionic surfactants functioned identically and went through the same mechanism to produce the cloud point phenomenon. The size and structure of the nonionic surfactant micelles are unclear, though. In order to determine the precise size and shape of amphiphilic micelles, DLS and SANS measurements were performed.

**Fig. 3:**
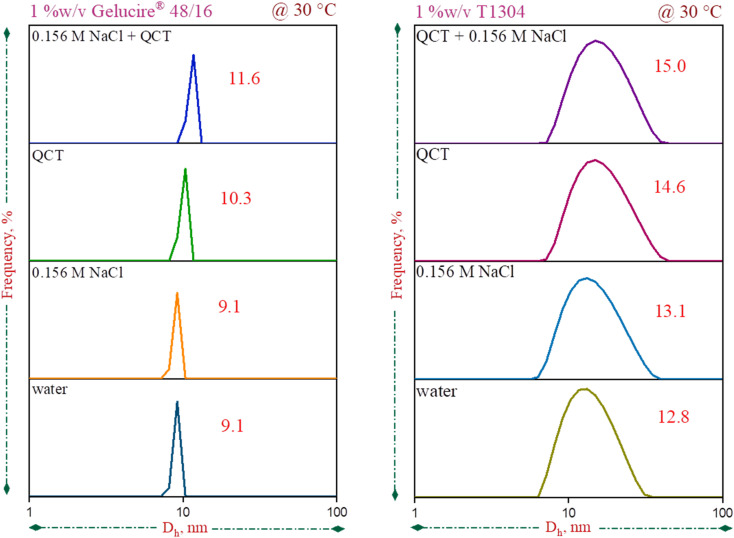
(a) Apparent hydrodynamic diameter of 1% w/v aqueous solution of (a) Ge 48/16 and (b) T1304.

DLS was used to analyse the size and distribution of the aggregates in the Ge 48/16 and T1304 micellar solutions under different conditions at 30 °C. The hydrodynamic diameter (*D*_h_) for Ge 48/16 solutions is depicted in Fig. 3(a). The *D*_h_ of pure 1% w/v aqueous solution of Ge 48/16 is nearly 9.1 nm which is comparable to the reported values^54^. In saline conditions, in the presence of 0.15 M NaCl, due to low concentration of salt, the value remains unchanged. For the solutions saturated with QCT, the *D*_h_ values do, however, marginally rise. When the saline solutions of Ge 48/16 are saturated with QCT molecules, the *D*_h_ value increases further to 11.6 nm. A similar tendency is also seen in the case of T1304, as shown in Fig. 3(b). The *D*_h_ value for T1304 in a 1% w/v aqueous solution is 12.8 nm. The micellar size in the saline state rises marginally to 13.1 nm. In the T1304 solution saturated with QCT, the size of the micelle increases to 14.6 nm in the absence of salt and further increases to 15.0 nm in saline environments. The significantly greater molecular weight and star-shaped structure of T1304 contribute for its larger micelle size. It is evident from the two cases that the micellar peak shifted to the higher *D*_h_ values, indicating that micellization as well as micellar growth were strongly favoured. It should be emphasised that there is very little change in saline conditions. Generally, the increase in micelle size with the addition of salt is related to its water structure making capability that makes the bulk solvent poorer for the solvation of surfactant molecules which induces the aggregation of surfactant units, promotes micellization and results in micellar growth. However, in the present case, in saline condition, with the concentration of salt as low as 0.15 M, the effect is not well pronounced resulting in minimum size change. It is interesting to note that, in both cases, the QCT encapsulation in the micellar core slightly elevated the micelle size (in the absence of salt). Nevertheless, the addition of salt besides improving the micelle size, dramatically increases QCT encapsulation. Hence, the improved QCT encapsulation and favoured micellization in the saline conditions leads to augmented micellar size. As a result, both of the nonionic surfactants show the greatest size change in the saline surfactant solutions saturated with QCT. Thus, it brings us to a conclusion that micellization is strongly favoured with improved QCT encapsulation in saline conditions.

**Fig. 4:**
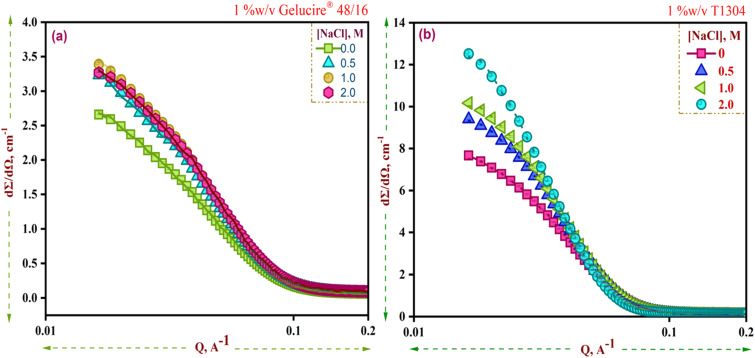
SANS curves for 1% w/v solutions of (a) Ge 48/16 and (b) T1304 in the presence and absence of salt at 30 °C.

The scattering profiles of 1% w/v Ge 48/16 and 1% w/v T1304 are shown in Fig. 4(a) and (b), respectively. In line with the DLS results, as evident in Fig. 4(a) and (b), salt significantly increased the scattering cross section of Ge 48/16, and T1304, respectively, and this tendency persisted as salt content was increased. As stated in the preceding section, the presence of salt in the micellar system enhanced hydrophobicity and encouraged micellization. Hence, as observed in Fig. 4, the addition of salt improves the scattering cross section, confirming micellar growth and the formation of larger micelles. This is explained by the fact that salt disrupts the hydration layers surrounding the PEG chains for both amphiphiles, reducing their total solubility in the process. As a result, bigger aggregates are formed as more and more surfactant units self-assemble. In other words, salt alters the hydrophile-lipophile balance of both nonionic surfactants and encourages micelle formation and growth.

The derived parameters shown in Table 1 further support this. According to SANS findings, spherical micelles are observed in both nonionic surfactants, and an increase in the concentration of salt affects the size of the micelles in both nonionic surfactants. In contrast to Ge 48/16, the impact is more prominent in T1304. Ge 48/16's core radius, *R*_c_, increases marginally, whereas T1304's micellar size significantly increases, possibly as a result of T1304's high molecular weight and a greater proportion of PEG constituents.

**Table 1:** SANS parameters for the micellar solutions of 1% w/v Ge 48/16 and 1% w/v T1304 in the presence and absence of salt

Polymer	[NaCl] (M)	Core radius *R*_c_ (Å)	Polydispersity, (*Φ*)	Morphology of micelles
1% w/v Ge 48/16	0	31.7	0.34	Spherical
	0.5	32.3	0.32	Spherical
	1.0	32.8	0.31	Spherical
	2.0	33.3	0.29	Spherical
1% w/v T1304	0	43.6	0.23	Spherical
	0.5	46.0	0.22	Spherical
	1.0	48.1	0.21	Spherical
	2.0	53.3	0.21	Spherical

**Fig. 5:**
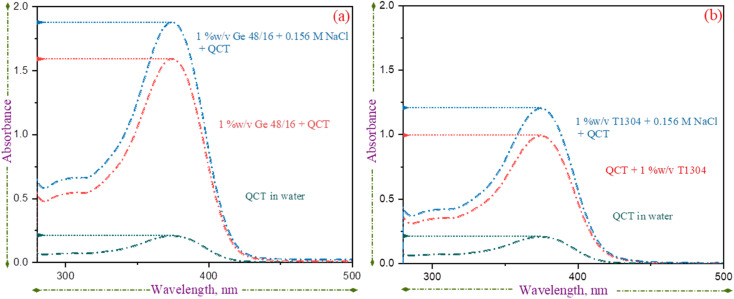
UV-vis absorbance spectra of QCT in 1% w/v aqueous solutions of (a) Ge 48/16 and (b) T1304.

The previous sections have shown that the introduction of QCT into the micellar system has an impact on its physicochemical properties. Specifically, the CP decreases slightly and the micelle size increases. To understand the interaction between QCT and surfactant molecules, UV absorption and FTIR was used. The UV spectra of QCT in water and surfactant solutions (with and without saline conditions) were studied and are presented in Fig. 5. As reported previously, the *λ*_max_ for QCT was found to be 373 nm.^58^ QCT has relatively low aqueous solubility, and in the absence of surfactants, a small, distinct peak is visible. However, in micellar solutions, the absorption peak becomes more prominent, indicating that more QCT molecules are encapsulated in the core of surfactant micelles. The presence of nonionic surfactants does not shift the peak values, but absorbance does increase, likely due to the increased encapsulation of QCT molecules. The extent of the increase in absorbance is specific to each surfactant's level of interaction with QCT.

Based on our analysis of Fig. 5(a) and (b), it seems that Ge 48/16 (with and without salt) exhibits a higher level of absorption. This can be attributed to the strong hydrophobic interactions between the palmitic (C_16_) and stearic (C_18_) acid chains in the Ge 48/16 structure with QCT molecules. In comparison, the interactions with the PPG core of T1304 is relatively weak, resulting in lower absorption values. However, the presence of salt in the solution intensifies the absorption peak in both cases, with Ge 48/16 still demonstrating dominance. As mentioned earlier, the addition of salt decreases the solvation of surfactant molecules, promoting micellization and allowing for greater accommodation of water-insoluble molecules. This improved encapsulation of QCT molecules leads to higher absorption values for both surfactants in the saline condition, as supported by our HPLC findings discussed in the following section.

**Fig. 6:**
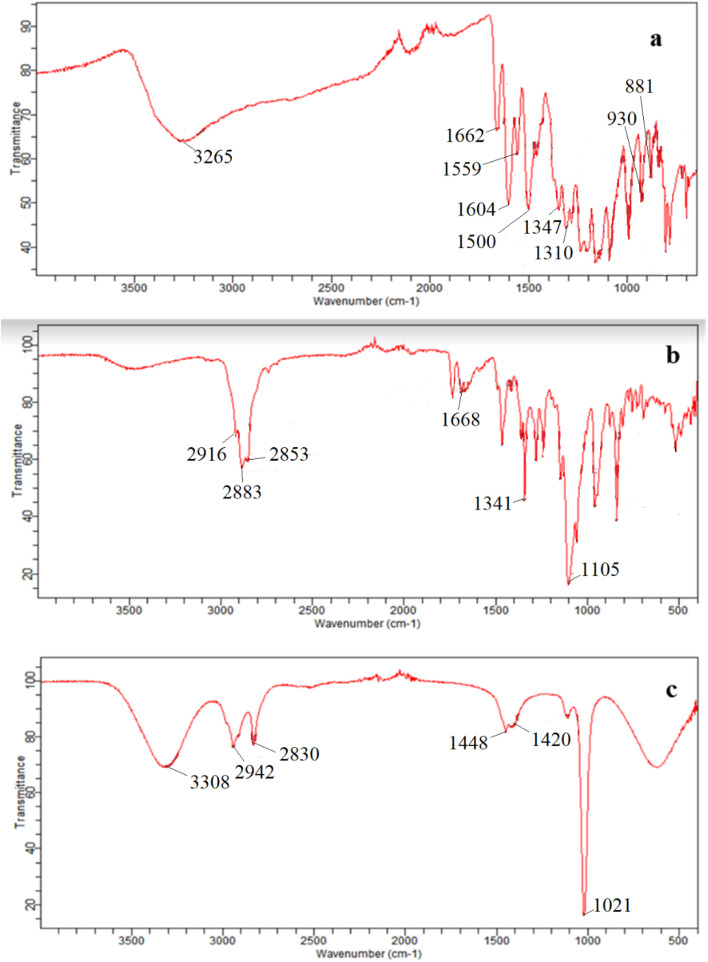
FT-IR spectral profile illustrating the favourable interaction of QCT with Ge 48/16.

To identify and have a better insight on the interaction between the quercetin and the surfactant micelles, the FTIR spectra of pure quercetin (QCT) (Fig. 6(a)), pure Ge 48/16 (Fig. 6(b)) and the quercetin encapsulated Ge 48/16 micelles (Fig. 6(c)) were compared. The FTIR measurements were restricted only to Ge 48/16 and were not performed for T1304, as there are several reports in literature confirming the existence of hydrophobic–hydrophobic interactions between QCT and PEG-PPG block copolymers using FTIR.^58^

The FTIR spectrum of pure quercetin is depicted in the Fig. 6(a), highlighting its distinctive spectral features. The OH group stretching vibrations were observed at 3265 cm^−1^, while the bending vibrations of the phenolic OH group appeared at 1347 cm^−1^. The absorption band corresponding to the C=O aryl ketonic stretch was seen at 1662 cm^−1^. The C=C aromatic ring stretching modes were seen at 1604 cm^−1^, 1559 cm^−1^, and 1500 cm^−1^. In addition, the in-plane bending band of C–H in the aromatic hydrocarbon appeared at 1310 cm^−1^, while the out-of-plane bending bands were seen at 930 cm^−1^, 881 cm^−1^, 840 cm^−1^, and 721 cm^−1^. Furthermore, bands at 1284 cm^−1^, 1209 cm^−1^, and 1161 cm^−1^ were attributed to the C–O stretching in the aryl ether ring, the C–O stretching in phenol, and the C–CO–C stretch and bending in ketone, respectively.^79^

When QCT is interacting with Ge 48/16 as depicted in Fig. 6(c), the QCT + Ge 48/16 systems exhibit a range of IR band shifts with varying degrees of intensity reduction. Specifically, the characteristic peak of the phenolic O–H group in the IR spectrum of QCT, which is typically observed at 3265 cm^−1^, weakens considerably in the QCT + Ge 48/16 system and undergoes a significant shift to 3308 cm^−1^. This indicates that the phenolic O–H group of QCT interacts with lipidic surfactant, which enhances its solubility. There is also a slight shift in the IR band position of other functional groups. The shift in the IR band position of QCT when combined with Ge 48/16, ranging from slight to strong, may be attributed to hydrophobic interaction and solubilization of QCT by the surfactant.

### 3.2 Solubilization of QCT in surfactant micelles

Surfactants are a sizable class of pharmaceutical excipients that serve as solubilizers, emulsifiers, foamers, wetting agents, *etc.* in a range of drug delivery systems.^80–82^ Surfactant molecules produce micelles colloidal aggregates with diverse microstructure and areas of varying polarity above the critical micelle concentration (CMC). The different levels of polarity in the micelles make it easier to incorporate drug molecules that aren't very water soluble, which causes solubilization and an increase in the drug's apparent aqueous solubility. Surfactants' ability to solubilize medicines in micellar form has been the focus of numerous studies.^1,32,83–89^ Several authors have examined the effects of polysorbates, ethoxylated alcohols, ethoxylated alkyl esters, alkyl sulphates, and alkyl trimethyl ammonium bromides (TABs) on the solubility of various drugs^90–98^ and observed that the micellar solubilization capacity of a surfactant depends largely on its architecture. Hence, in the present study we have investigated the solubilising behaviour of two PEG based nonionic surfactants, Ge 48/16 and T1304 to check the solubility power of both the nonionic surfactants for the poorly water-soluble anticancer drug, QCT in the presence and absence of salt using HPLC technique.

**Fig. 7:**
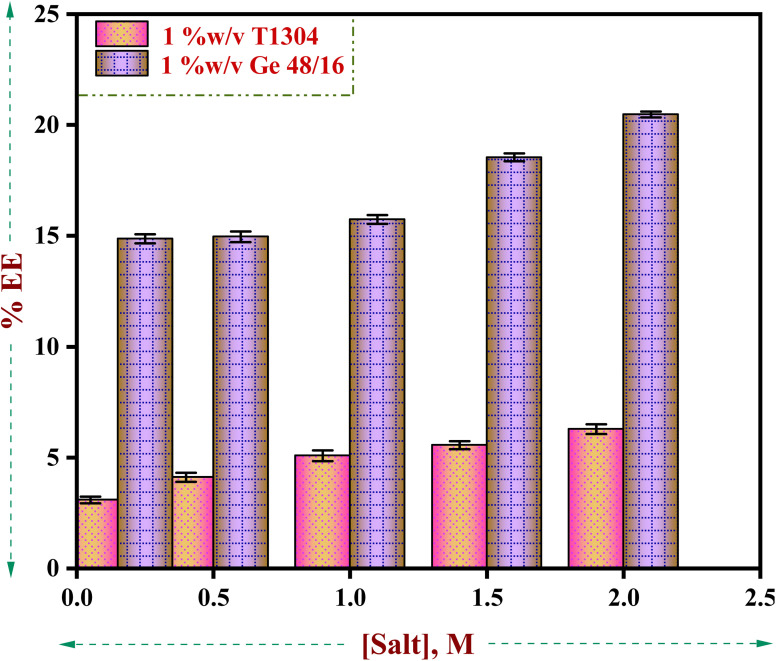
Drug loading amount/solubility (mg mL^−1^) of QCT in 1% w/v aqueous solutions of Ge 48/16 and T1304 as a function of salt concentration.

As evident in Fig. 7 and Table 2, the solubilising capacity of Ge 48/16 in the presence and absence of salt is many folds greater than T1304 and a similar trend is noted in the presence of salt. The DLA and EE% of both the nonionic surfactants remained low in the absence of salt. However, with subsequent increase in the concentration of salt from 0.156 M to 2.0 M, a gradual rise in DLA and EE% can be observed. This increase in the encapsulation capacity of both the amphiphiles can be correlated with the salting out action of NaCl as discussed in the previous sections. The increase in the concentration of salt promoted aggregation due to the well-known salting out action. A more hydrophobic environment is created for the surfactant units resulting in increased participation in micelle formation. Consequently, larger micelles with more hydrophobic room to accommodate the hydrophobic molecule, QCT is created. Hence, the encapsulation efficiency significantly improves in the presence of salt. The dominance of Ge 48/16 in solubilizing QCT molecules can be attributed to the strong hydrophobic interactions existing between palmitic (C_16_) and stearic (C_18_) acids chains with the QCT molecules.

**Table 2:** Drug loading amount and entrapment efficiency of QCT in presence of NaCl with Ge 48/16, T1304 and mixed micellar systems at 30 °C

[NaCl] (M)	1% w/v Ge 48/16		1% w/v T1304	
	EE (%)	DLA (mg)	EE (%)	DLA (mg)
0.0	11.8	0.59	1.86	0.093
0.156	14.87	0.74	3.08	0.154
0.5	14.96	0.75	4.11	0.205
1.0	15.74	0.79	5.08	0.254
1.5	18.54	0.93	5.56	0.278
2.0	20.48	1.02	6.28	0.314

In conclusion, our results show that Ge 48/16 outperforms T1304, exhibiting a significant solubilizing potential for QCT in both the presence and absence of salt. Furthermore, the salting out effect, which produces larger and more hydrophobic micelles that can more easily incorporate QCT molecules, contributes to a significant improvement in the encapsulation efficiency of both nonionic surfactants. These results offer important information on the possible use of Ge 48/16 as a powerful carrier for hydrophobic substances like QCT in a variety of applications.

## 4. Conclusion

A comprehensive study was conducted on Ge 48/16 and T1304, two biocompatible excipients based on PEG amphiphiles, to better understand their behavior in the presence and absence of salt. Various techniques such as cloud point, dynamic light scattering, small-angle neutron scattering, Fourier transform infrared spectroscopy, UV spectrophotometry, and high-performance liquid chromatography were employed. The study revealed that both self-assembling systems have a similar mechanism for the cloud point phenomenon, where hydrogen bonds between PEG chains and water are broken as temperature rises, leading to amphiphilic units' limited solubility in water. However, the presence of NaCl significantly decreased the cloud point of both amphiphiles due to its salting out action. DLS and SANS measurements showed that NaCl had a comparable effect on the micellar size of both nonionic surfactants, with T1304 experiencing a more pronounced increase than Ge 48/16. Despite salt improving the solubilizing properties of both surfactants, Ge 48/16 performed better than T1304. FTIR and UV spectrophotometry revealed a significant hydrophobic interaction between the QCT molecules and the micellar core's hydrophobic chains. Based on the study's findings, Ge 48/16 may be utilized as nanocarriers for better therapeutic outcomes.

## Conflicts of interest

There are no conflicts to declare.

## Supplementary Material
